# Efficacy of a cardiac rehabilitation program in a municipal sports center compared to the hospital program: randomized controlled trial eCARCEX

**DOI:** 10.23938/ASSN.1050

**Published:** 2023-11-24

**Authors:** Juan Izquierdo-García, Adrián Arranz-Escudero, Rocío Tello de Meneses, Noelia de la Torre, Isabel M. Amat-Macías, Juan I. Castillo Martín, M. Paz Sanz-Ayán, Guillermo Moreno

**Affiliations:** 1 Universidad Complutense de Madrid Complutense University of Madrid Faculty of Nursing, Physiotherapy and Podiatry Department of Radiology, Rehabilitation and Physiotherapy Madrid Spain; 2 Madrid Health Service Hospital Universitario 12 de Octubre Rehabilitation Service Madrid Spain; 3 Madrid Health Service Hospital Universitario 12 de Octubre Cardiology Department Madrid Spain; 4 Madrid City Council Plata y Castañar Municipal Sports Center Madrid Spain; 5 Universidad Complutense de Madrid Complutense University of Madrid Faculty of Medicine Department of Radiology, Rehabilitation and Physiotherapy Madrid Spain; 6 Universidad Complutense de Madrid Complutense University of Madrid Faculty of Nursing, Physiotherapy and Podiatry Department of Nursing Madrid Spain

**Keywords:** Cardiac Rehabilitation, Telerehabilitation, Exercise Therapy, Health Education, Acute Coronary Syndrome, Rehabilitación Cardiaca, Telerehabilitación, Terapia por Ejercicio, Educación en Salud, Síndrome Coronario Agudo

## Abstract

**Background:**

This study aimed to analyze the effects of an outpatient cardiac rehabilitation program in a municipal sports center on functional capacity and adherence to physical activity - among other variables - compared to an in-hospital program.

**Methods:**

Randomized clinical trial that included two parallel groups of acute coronary syndrome patients who performed a cardiac rehabilitation program that consisted of moderate physical exercise intervals along with learning healthy habits in a municipal sports center (EG) and in a tertiary hospital (CG) between September 2019 and June 2020. We collected the following data: compliance, anthropometrical, clinical, psychological variables, diet and tobacco habits, strength and functional capacity from ergospirometry.

**Results:**

Twenty-two patients completed the cardiac rehabilitation program (EG=12, CG=10). Significant improvement was observed for cholesterol, the sit-and-stand test, cardiac frequency in VT1 and VT2, and watts in VT1 in the CG, and for HDL-cholesterol, triglycerides, the sit-and-stand test, and frequency, and watts in VT1 in the EG. Better achievement was found in the CG for cardiac frequency in VT2 (11.17 vs 2.88 bpm) and in EG for HDL-cholesterol (11.0 vs 0.63 mg/dL).

**Conclusions:**

We are unable to determine the effectiveness of the out-of-hospital cardiac rehabilitation program due to a lack of power (high number of withdrawals caused by COVID-19 lockdown). However, the EG achieved higher HDL-cholesterol levels, while cardiac frequency in VT2 was higher in the CG.

## Resumen

**Fundamento:**

El objetivo de este estudio es analizar los efectos de un programa de rehabilitación cardiaca (PRC) extrahospitalario en un centro municipal deportivo sobre la capacidad funcional y la adherencia a la actividad física, entre otras variables, en comparación con un modelo hospitalario.

**Métodos:**

Ensayo clínico aleatorizado con dos grupos paralelos de pacientes con síndrome coronario agudo que realizaron un PRC con ejercicio físico moderado interválico coordinado con educación en hábitos saludables en un centro deportivo municipal (GE) y en un hospital terciario (GC), entre septiembre de 2019 y junio de 2020. Se analizaron variables de adherencia, antropométricas, clínicas, psicológicas, de fuerza, de prevención secundaria (dieta, tabaquismo) y capacidad funcional con la prueba de ergoespirometría.

**Resultados:**

Veintidós pacientes completaron el PRC (GC=10, GE=12). Se observaron mejoras significativas pre-post en GC (colesterol, test de la silla, frecuencia cardiaca en VT1 y VT2, y vatios en VT1) y en GE (colesterol HDL, triglicéridos, test de la silla, y frecuencia cardiaca y vatios en VT1). Estas mejoras fueron mayores en el GC para la frecuencia cardiaca en VT2 (11,17 vs 2,88 lpm) y en el GE para el colesterol HDL (11,0 vs 0,63 mg/dL).

**Conclusiones:**

Este estudio no ha podido determinar la eficacia de los PRC extrahospitalarios por falta de potencia (abundantes abandonos debidos al confinamiento por COVID-19). A pesar de ello, en el GE se observó mayor aumento en colesterol HDL que en el GC, aunque la frecuencia cardiaca en VT2 fue mayor en el GC.

## INTRODUCTION

Cardiovascular disease continues to be the leading cause of death in Spain, despite the fact that in recent decades there has been a sustained trend towards lower incidence and mortality^1^^,^^2^. At the European level, it accounts for 50% of health expenditure and 25% of productivity losses^3^^,^^4^.

The implementation of therapeutic options, such as cardiac rehabilitation programs (CRPs), has demonstrated broad benefits on cardiovascular morbidity and mortality, functional capacity, obesity, cardiovascular risk factors, and quality of life^3^^,^^5^. In addition, clinical practice guidelines recommend CRPs in patients with acute coronary syndrome (I-A recommendation level)^4^ and in patients with heart failure (I-A recommendation level)^6^.

However, despite the benefits of CRP, its implementation in clinical practice is scarce. According to the European registry EUROASPIRE-V, only 46% of patients after acute coronary syndrome were recommended to participate in a CRP and, of these, only 69% of the sessions (32% of the total number of patients) performed at least half of the sessions^7^.

In Spain, around 30% of patients referred from discharge do not complete the CRP^5^. One of the main reasons for dropout is the delay in starting the physical exercise program (PEP) since, for each day of delay, adherence to the CRP decreases by 1%^5^. As a matter of fact, and with the intention of reducing this waiting time in patients with acute coronary syndrome, cardiac telerehabilitation programs have been proposed, with out-of-hospital performance of the PEP, using public resources and new information and communication technologies for remote monitoring of activity^8^.

Several models of cardiac telerehabilitation have been studied, using different electronic platforms and different levels of supervision and monitoring^9^^,^^10^. Cardiac telerehabilitation has been found to be safe in patients with low- and moderate-risk coronary artery disease, and as effective as hospital CRP for the control of risk factors and increased functional capacity, showing even better results in terms of adherence^11^^-^^13^. Cardiac telerehabilitation at home has been shown to improve patients’ quality of life, as well as physical parameters such as functional capacity, physical activity habits,^14^ heart rate or maximal oxygen consumption^15^.

However, we do not have at our disposal studies carried out in rehabilitation settings other than home or hospital settings, such as municipal sports centers, which could be a useful alternative. Therefore, more studies with high methodological quality analyzing and comparing the effects of cardiac telerehabilitation programs in out-of-hospital centers with hospital-based CRPs are needed in our medium to gain an in-depth understanding of the effects of cardiac telerehabilitation.

Therefore, the aim of this study was to compare the effects of an out-of-hospital CRP performed in a municipal sports center versus a hospital CRP in patients with acute coronary syndrome on adherence to physical activity, anthropometric, clinical, and psychological variables, strength, heart-healthy habits and functional capacity in ergospirometry.

## SUBJECTS AND METHODS

### Design

Randomized, controlled, open-ended clinical trial with two parallel (1:1) treatment groups, conducted at the Multidisciplinary Cardiac Rehabilitation Unit (MCRU) of the Hospital Universitario 12 de Octubre (tertiary hospital of Madrid, Spain) between September 2019 and June 2020. The recommendations of the CONSORT (*CONsolidated Standards Of Reporting Trials*) guidelines were taken into account at all times.

### Participants

All patients with acute coronary syndrome referred to UMRC during the study period were selected. The inclusion criteria were patients over 18 years of age diagnosed with low- and moderate-risk acute coronary syndrome^16^, physically able to exercise, with basic use of applications on a smartphone, and willing to give written informed consent. Patients with baseline ergospirometry outcome below the maximality criteria (respiratory quotient >1.1)^17^ with mental disability or with comorbidities that prevent PEP from being performed were excluded.

After applying the selection criteria, the patients who signed the informed consent form were randomized to two groups in a 1:1 ratio using the “RANDOM. BETWEEN” (Microsoft Excel®). Both groups received the same intervention, so there were no different attention times, exercises, or recommendations; only the environment changed: the control group carried out a hospital CRP (at the MCRU) and the intervention group carried out an extra-hospital CRP (at the *Plata y Castañar* Municipal Sports Center). Due to the characteristics of the center (waiting list), patients in both groups started the CRP six months after hospital discharge.

Patients who refused to participate or withdrew informed consent underwent the center’s conventional cardiac rehabilitation program.

### Study protocol and intervention

The intervention protocol in both groups consisted of 16 sessions of physical exercise, two days a week, for eight weeks, based on: 30 minutes of callisthenic exercises of the column, upper and lower limbs, including plyometric exercises and muscle strength exercise; 30 minutes of interval aerobic endurance exercise on a moderate-intensity cycle ergometer (between the aerobic ventilatory threshold [VT1] and the anaerobic ventilatory threshold [VT2] of ergospirometry, and achieving an exertion perception score of 5-6 on the modified Borg scale), and ending with five minutes of flexibility exercises.

In both groups, all sessions were carried out under the supervision of a physiotherapist specializing in cardiac rehabilitation, who is part of the research team. In addition, in the control group they were supervised by a rehabilitation specialist from the research team, and in the intervention group by a specialist in Sports Medicine.

In addition, combined with the exercise program, weekly 30-minute health education (HE) group workshops were offered in hospital classrooms. These workshops are held on a regular basis during the hospital’s CRP, which are coordinated by cardiac rehabilitation-specialized nurses. They consist of information on acute coronary syndrome: recommendations for physical exercise, smoking, nutrition, cardiovascular risk factors, heart-healthy habits, emotional stress, sexual dysfunctions, and social and technological resources. They are taught by cardiologists, pulmonologists, urologists, nurses, psychologists, social workers, rehabilitation physicians and physiotherapists. Access is allowed to all patients referred from the hospital or from other facilities. Both groups (control and experiential) attended the same workshops taught with the same methodology.

Once the CRP was completed, in the final consultation of this phase, the rehabilitation physician prescribed physical exercise guidelines (type of exercise, duration, intensity and frequency) adapted to the chronic patient to ensure continuity of care^3^.

To monitor patients belonging to the out-of-hospital CRP in a sports center (experimental group), a monitoring device, S-PATCH3-Cardio, wearable and linked to a mobile phone, was used that monitors the heart rhythm in real time, records possible cardiac events and performs remote telemetry during exercise; consultations were scheduled in case of adverse events or patient need. In addition, patients in the out-of-hospital CRP had to perform a 24-hour drill to learn how to use the monitoring device. A wireless, closed, integrated, workstation-controlled cardiac rehabilitation system was used to monitor patients in the hospital CRP (control group). The wireless monitoring of the system was executed through the *“*Sana sprint” software (Ergoline), which monitors electrocardiographic rhythm, heart rate, oxygen saturation and blood pressure.

### Variables

At the patient’s first visit, at the beginning of the CRP, an initial evaluation was performed that included:

- Demographic variables: age (years), sex (male, female).
- Review of pharmacological treatment: use of antiplatelet drugs (acetylsalicylic acid [ASA] and P2Y12 inhibitors: ticagrelor, prasugrel and clopidogrel), lipid-lowering drugs (statins), drugs for blood pressure and heart rate control (angiotensin-converting enzyme [ACE], angiotensin receptor blockers [ARBs] and beta-blockers), nitrates and diuretics.
- Anthropometric measurements: weight (kilograms), height (meters), body mass index (BMI) (kg/m^2^) and abdominal circumference (centi-meters).
- Laboratory parameters: total cholesterol (mg/dL), LDL cholesterol (mg/dL), HDL cholesterol (mg/dL), triglycerides (mg/dL), blood glucose (mg/dL), and glycosylated hemoglobin (HbA1c) (%).
- Clinical variables: type of acute coronary syndrome (ACS), ST-segment elevation (STEACS) or non-ST-segment elevation (NSTEACS); coronary artery bypass therapy (percutaneous coronary intervention or coronary artery bypass surgery), number of coronary vessels involved (one, two, or three, or more than three), stroke volume or beat-to-heart volume (SV) (mL), left ventricular end-diastolic volume (EDV) (mL), ejection fraction (SV×100/EDV) (%), blood pressure (mm Hg), and smoking (yes/no).
- Upper body muscle strength assessed with manual grip strength using CAMRY EH101 dynamometer (*hand grip test*); normal values: men aged 45-60 years: 11.3-55.8; men >60 years: 6.6-50.8; women aged 45-60 years: 1.1-23.9; women >60 years: 1-23,2^18^.
- Muscle strength and endurance of the lower body assessed using the 30 second chair stand test (30s-CST), which consists of asking participants to sit in a chair without armrests, placing their arms cross, and complete the maximum number of squats in 30 seconds. Only squats in which the participant makes a gluteal-chair contact (without sitting) and fully extends the knees when standing up will be counted. Normal values: men <65 years: 14-19 squats; men 65-69 years: 12-18; men 70-74 years: 12-17; women aged <65 years: 12-17; women aged 65-69 years: 11-16; women aged 70-74 years: 10-15 squats^19^.

Various questionnaires on secondary prevention measures were also administered:

- Adherence to the Mediterranean diet with the Mediterranean Diet Adherence Screener (MEDAS) questionnaire by Schröder et al.^20^. Each of its 14 items is rated with 0 or 1 according to the diet pattern, referring to daily or weekly consumption, during the last three months. The score ranges from 0 to 14 points; The higher the score, the greater the adherence
- Tobacco dependence: assessed using the Fagerstrom Test, a six-item scale that assesses people’s nicotine dependence. The higher the score on the scale, the higher the dependency^21^.
- Motivation to quit smoking: assessed using the Richmond Test. It consists of four items, item 1 scores from 0 to 1 and the rest from 0 to 3. The score range ranges from 0 to 10; The higher the score, the greater the motivation to quit smoking^22^.
- Symptomatology of anxiety and depression: the Hospital Anxiety and Depression Scale (HADS) by Zigmond and Snaith^23^^)^ was used, composed of two subscales, one for anxiety (HADS-A) and the other for depression (HADS-D), with seven items each, which are answered with a four-point Likert scale (0 to 3). The range is from 0 to 21 in each subscale, and from 11 points onwards, cases of anxiety or depression are identified; Results between 8 and 10 are classified as suspected of anxious or depressive symptomatology, and below 8 (from 0 to 7) it is considered that there is an absence of symptoms^23^.

The main variables studied were:

- Adherence to physical activity: using the International Physical Activity Questionnaire (IPAQ) by Craig et al.^24^. It consists of five questions about the frequency, duration, and intensity (vigorous or moderate) of physical activity in the last week (last seven days). It also includes the frequency and duration of the walking activity and the time the person sits on a weekday. It allows individuals to be classified into three categories (low, moderate, vigorous) according to the estimated energy expenditure for each activity: vigorous, ≥8 MET (metabolic equivalent task); moderate, 4-3.3 METs; and low, ≤3.3 MET^24^.
- Functional capacity: conducting an ergospirometry^25^ obtaining: test duration time (minutes), oxygen consumption (VO_2_, normal value: men aged 50-59 years: 20.2-35.7 (mL/kg/min); men ≥60 years: 17.5-31.4; women aged 50-59 years: 26.1-45.3; women ≥60 years: 20.5-44.2), heart rate (HR) in beats per minute (bpm, normal range: 50-100) and power (watts, W) generated in VT1 and VT2, and maximum VO_2_ values reached (VO_2_ maximum, normal range: men aged 50-59 years: 18-43 mL/kg/min; men >60 years: 16-41; women aged 50-59 years: 15-38; women >60 years: 13-35) and HR in bpm (HRmax, normal range >90), and power (watts, no normative ranges)^26^^,^^27^.

Adherence to the program was measured by the number of sessions performed in each group.

At the end of the eight weeks of the CRP, the same measurements were taken again to evaluate the effectiveness of the intra- and inter-group program.

### Sample size calculation

With the intention of achieving 71% adherence to the hospital CRP and 95% to the extra-hospital CRP, for a confidence level of 95% and a statistical power of 80%, a sample size of 76 participants (38 per branch) was estimated. The Epidat 4.1® tool was used to calculate the sample size.

### Statistical analysis

Continuous variables are presented as mean and standard deviation (SD) and qualitative variables as absolute frequencies and percentages. The normality of the variables was contrasted with the Shapiro-Wilk test. To compare means between the initial and final evaluation moments and between both groups, the Student’s t-test was used, for paired or independent samples, respectively. Categorical variables were compared between independent groups using the Chi-Square test (χ2) or Fisher’s exact test according to the presence of frequencies less than 5, and between dependent groups using the McNemar test. In addition, the magnitudes of the differences were calculated with the effect sizes of the means (d), following Cohen’s criteria to determine its magnitude: small (0.2-0.49), medium (0.5-0.79) or large (>0.8). The level of statistical significance was set at 5%. All analyses were performed with the SPSS version 25.0 (IBM, United States).

### Ethical approval and informed consent

The study was approved by the Clinical Research Ethics Committee of the Hospital Universitario 12 de Octubre (CEIm internal approval number: 19/176). The ethical principles of biomedical research of the Declaration of Helsinki (revised in 2013), the principles of the International Conference on Harmonization (ICH) on Good Clinical Practices, and the principles of the Biomedical Research Act (14/2007) were complied with at all times. Prior to inclusion in the study, all participants were informed of the objectives of the research and gave their written informed consent. The prototype of the study was recorded in ClinicalTrials.gov coded NCT04121702.

## RESULTS

**Figure 1:**
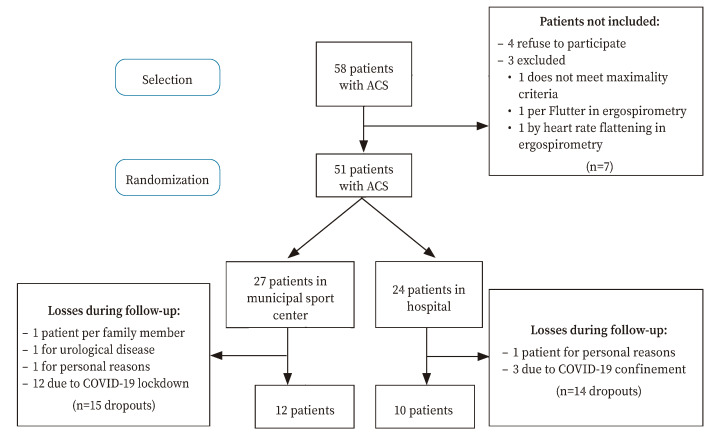
Study flow chart

Of the 58 selected patients with ACS, 51 were included; 27 were randomized in the out-of-hospital CRP group and 24 in the hospital CRP group. During the follow-up of the study, 29 patients were excluded due to abandonment of the PEP, 86.2% due to the confinement measures and restrictions caused by COVID-19. As a result, the 22 patients who completed the entire study protocol were analyzed, 10 in the hospital CRP group and 12 in the out-of-hospital CRP group (Fig. 1).

The two randomization groups were similar at baseline in relation to sociodemographic and clinical variables, risk factors, cardiovascular history, and pharmacological treatment (Table 1). The group with out-of-hospital CRP was younger and had a lower frequency of dyslipidemia, although not significantly. None of the patients showed involvement in three vessels or atrial fibrillation.

**Table 1:** Comparison of baseline characteristics of patients in both randomization groups at baseline of the Cardiac Rehabilitation Program

	Cardiac Rehabilitation Program				p
	Hospital		Municipal sport center		
	n = 10		n = 12		(χ^2^)
*Sociodemographic variables*					
Age, *mean* (*DE*)	61.3	(6.13)	55.25	(10.26)	0.118^a^
Sex (male), *n (%)*	8.0	(80.0)	11.00	(91.7)	0.622
*Clinical variables, n (%)*					
SCA type (SCASEST)	5	(50.0)	7	(58.0)	0.700
Affected vessels (one)	8	(80.0)	7	(64.0)	0.410
FEVI, *mean* (*SD*)	56.2	(5.2)	59.6	(9.1)	0.320^a^
*Risk factors and background, n (%)*					
Obesity	1	(10.0)	1	(8.3)	0.890
Dysslipemia	9	(90.0)	7	(58.3)	0.097
Type 2 diabetes mellitus	2	(20.0)	1	(8.3)	0.430
High blood pressure	4	(40.0)	5	(41.7)	0.940
Heart failure	0	-100	0	-100	1
Active smoker	6	(66.7)	8	(80.0)	0.510
*Pharmacological and invasive treatment, n (%)*					
ARA-II	0	(0.0)	1	(8.3)	0.350
IECA	5	(50.0)	8	(66.6)	0.430
Beta blockers	6	(60.0)	6	(50.0)	0.640
Statins	10	-100	12	-100	1
Antiplatelet agents					
Ticagrelor	8	(80.0)	7	(58.3)	0.280
Prasugrel	1	(10.0)	3	(25.0)	0.360
Clopidogrel	0	(0.0)	1	(8.3)	0.350
ASA	10	-100	12	-100	1
Percutaneous coronary intervention (PCI)	10	-100	12	-100	1

a: Student’s t-test; SD, standard deviation; ASA: acetylsalicylic acid; ARBs: angiotensin II receptor antagonists; LVEF, left ventricular ejection fraction; ACE inhibitors: angiotensin-converting enzyme inhibitors; NSTEACS: non ST-elevation acute coronary syndrome.

No baseline differences were observed between groups in terms of level of physical activity, anthropometric variables, muscle strength, and functional capacity, except for a mean HDL cholesterol level of 36.5% higher in the hospital CRP group (32.30; SD: 5.08 vs 44.11; SD: 13.61; p=0.020). There were also no differences in baseline secondary prevention measures, Mediterranean diet, tobacco dependence, and motivation to quit smoking. The frequency of anxiety in the control group was three times higher than in the out-of-hospital CRP group (55.6 vs 16.7%; p=0.022), which were the only significant differences observed at baseline with respect to psychological measures (Table 2).

**Table 2:** Comparison of baseline characteristics of patients at the beginning of the Cardiac Rehabilitation Program

	Cardiac Rehabilitation Program				p
	Hospital		Municipal sport center		
	n = 10		n = 12		(t-test)
*Clinical and anthropometric variables, mean (SD*					
Heart rate (lpm),	64.10	(14.38)	64.73	(8.90)	0.905
Systolic blood pressure (mm Hg)	113.60	(9.76)	119.82	(15.30)	0.286
Weight (kg)	77.38	(12.78)	83.55	(13.81)	0.313
Size (m)	1.68	(0.11)	1.70	(0.09)	0.369
BMI (kg/m^2^)	27.37	(3.90)	27.86	(1.87)	0.726
Abdominal circumference (cm)	98.20	(11.43)	98.89	(6.24)	0.875
*Biochemical parameters, mean (SD*					
Total cholesterol (mg/dL)	138.40	(28.81)	121.40	(25.49)	0.179
LDL (mg/dL)	73.44	(20.59)	61.00	(20.10)	0.200
HDL (mg/dL)	44.11	(13.61)	32.30	(5.08)	0.020
Triglycerides (mg/dL)	88.30	(26.60)	141.50	(90.51)	0.091
Glucose (mg/dL)	112.20	(20.84)	102.09	(20.12)	0.272
% Glycated hemoglobin	5.63	(0.95)	5.45	(0.06)	0.727
*Psychological variables (HADS), n (%)*					
Anxiety	5	(50.0)	2	(16.7)	0.022^a^
Depression	1	(10.0)	0	0.00	0.323^a^
*Questionnaires (score), mean (SD*					
Fagerström	0.00	(0.00)	0.67	(1.78)	0.277
Richmond	0.00	(0.00)	1.58	(3.70)	0.218
MEDAS	8.78	(2.05)	9.92	(2.02)	0.219
Muscle strength (kg)					
right	42.61	(13.04)	45.21	(11.62)	0.636
left	40.63	(11.98)	41.78	(11.40)	0.825
30s-CST, *n (%)*	16.00	(4.72)	16.17	(4.67)	0.937
Functional capacity*, mean (SD)					
Time (min)	8.98	(2.76)	10.28	(2.81)	0.328
VO_2_ (mL/kg/min)					
in VT1	10.38	(2.31)	13.17	(8.32)	0.371
in VT2	15.85	(3.68)	19.08	(8.79)	0.344
maximum	18.30	(3.38)	22.66	(9.19)	0.220
HR (bpm)					
in VT1	77.88	(6.79)	86.18	(17.81)	0.229
in VT2	96.88	(13.38)	112.45	(21.71)	0.091
maximum	116.13	(18.72)	124.82	(20.89)	0.363
Power (W)					
in VT1	44.50	(13.86)	65.73	(55.64)	0.309
in VT2	77.75	(19.47)	121.91	(80.71)	0.151
maximum	102.75	(26.62)	143.91	(82.38)	0.194
*Physical activity level (IPAQ), n (%)*					
Low	1	(10.00)	0	(0.00)	0.104
Moderate	8	(80.00)	8	(66.7)	
Vigorous	0	(0.00)	4	(33.3)	

A: Chi-square test; *: ergospirometry; SD: standard deviation; HR: heart rate; HADS: Hospital Anxiety and Depression Scale; HDL: high-density lipoprotein; IPAQ: International Physical Activity Questionnaire; bpm: beats per minute; LDL, low-density lipoprotein; MEDAS: questionnaire on adherence to the Mediterranean diet; CRP: cardiac rehabilitation program; 30s-CST: 30 second chair stand test; VO_2_: oxygen consumption; VT1, aerobic threshold; VT2: anaerobic threshold.

The mean number of sessions attended was similar in both groups: 14.7 (SD: 1.3) in the hospital CRP group and 14.9 (SD: 2.7) in the outpatient CRP group (p = 0.90).

After the intervention sessions, both groups showed some significant differences with respect to their baseline situation (Table 3): the hospital CRP group decreased the mean total cholesterol (24.07 mg/dL) and increased the number of repetitions in the chair test (5.86), while the out-of-hospital CRP group increased HDL (11.9 mg/dL) and the number of repetitions in the chair test (4.16), reducing the basal triglyceride level by 33.1% (46.9 mg/dL).

Regarding the main variables of the study, neither group showed significant differences in either the levels of physical activity or the variables of oxygen consumption. Regarding the functional capacity variables in the ergospirometry test, the hospital CRP group increased heart rate in both the aerobic and anaerobic threshold (14.4% in VT1 and 11.2% in VT2) and power in VT1 (34.3%), while in the out-of-hospital CRP group, the mean heart rate and power at the aerobic threshold in VT1 increased (9.1% and 33.7%, respectively) (Table 3).

**Table 3:** Evolution of the parameters evaluated according to the group studied and intragroup comparisons

	Out-of-hospital CRP (n = 10)				p (paired Student’)	Cohen’s d
	Initial		Final			
*Clinical and anthropometric variables, mean (SD)*						
Heart rate (bpm),	64.10	(14.38)	56.88	(4.49)	0.303	
Systolic blood pressure (mm Hg)	113.60	(9.76)	121.13	(13.74)	0.071	
Weight (kg)	77.38	(12.78)	77.49	(13.00)	0.403	
Perímetro abdominal (cm)	98.20	(11.43)	97.93	(9.57)	0.619	
*Biochemical parameters, mean (SD)*						
Total cholesterol (mg/dL),	138.40	(28.81)	114.33	(18.10)	0.048	0.78
LDL (mg/dL)	73.44	(20.59)	49.11	(11.98)	0.051	
HDL (mg/dL)*)*	44.11	(13.61)	45.22	(15.42)	0.808	
Triglycerides (mg/dL)	88.30	(26.60)	100.22	(48.03)	0.469	
Glucose (mg/dL)	112.20	(20.84)	106.44	(22.86)	0.278	
% Glycated hemoglobin	5.63	(0.95)	5.98	(0.65)	0.035	
*Psychological variables (HADS), n (%)*						
Anxiety	5	(50.00)	1	(14.30)	0.350	
Depression	1	(10.00)	0	(0.00)	0.103	
*Questionnaires (score), mean (SD)*						
Fagerström	0.00	(0.00)	0.00	(0.00)	-	
Richmond	0.00	(0.00)	1.43	(3.78)	0.356	
MEDAS	8.78	(2.05)	9.43	(1.51)	0.383	
Muscle strength (kg)						
right	42.61	(13.04)	42.04	(10.99)	0.268	
left	40.63	(11.98)	39.77	(11.76)	0.528	
30s-CST, *n (%)*	16.00	(4.72)	21.86	(5.11)	0.012	1.35
Functional capacity*, mean (SD)						
Time (min)	8.98	(2.76)	10.71	(5.21)	0.456	
VO_2_ (mL/kg/min)						
in VT1	10.38	(2.31)	10.70	(3.12)	0.383	
in VT2	15.85	(3.68)	15.26	(4.78)	0.879	
maximum	18.30	(3.38)	16.45	(5.30)	0.267	
HR (bpm)						
in VT1	77.88	(6.79)	86.13	(8.66)	0.004	
in VT2	96.88	(13.38)	107.75	(15.51)	0.031	
maximum	116.13	(18.72)	116.50	(15.04)	0.308	
Power (W)						
in VT1	44.50	(13.86)	59.75	(23.68)	0.045	
in VT2	77.75	(19.47)	101.00	(30.43)	0.063	
maximum	102.75	(26.62)	114.25	(29.73)	0.147	
*Physical activity level (IPAQ), n (%)*						
Low	2	(20.00)	0	(0.00)	0.714	
Moderate	8	(80.00)	5	(71.40)		
Vigorous	0	(0.00)	2	(28.60)		
	*Out-of-hospital CRP (n = 12)*				*p*	*Cohen’s*
	*Initial*		*Final*		*(paired Student’)*	*d*
*Clinical and anthropometric variables, mean (SD)*						
Heart rate (bpm),	64.73	(8.90)	63.00	(14.18)	0.493	
Systolic blood pressure (mm Hg)	119.82	(15.30)	130.17	(8.84)	0.409	
Weight (kg)	83.55	(13.81)	84.33	(14.12)	0.900	
Abdominal circumference (cm)	98.89	(6.24)	97.00	(4.73)	0.800	
*Biochemical parameters, mean (SD)*						
Total cholesterol (mg/dL),	121.40	(25.49)	119.80	(22.40)	0.545	
LDL (mg/dL)	61.00	(20.10)	56.60	(12.90)	0.219	
HDL (mg/dL)	32.30	(5.08)	44.20	(3.35)	0.013	1.9
Triglycerides (mg/dL)	141.50	(90.5)	94.60	(42.26)	0.036	1.39
Glucose (mg/dL)	102.09	(20.12)	101.80	(9.76)	0.957	
% Glycated hemoglobin	5.45	(0.06)	5.47	(0.25)	0.205	
*Psychological variables (HADS), n (%)*						
Anxiety	2	(16.70)	3	(14.30)	0.392	
Depression	0	(0.00)	0	(0.00)	1	
*Questionnaires (score), mean (SD)*						
Fagerström	0.67	(1.78)	0.58	(1.73)	0.339	
Richmond	1.58	(3.70)	1.17	(2.86)	0.339	
MEDAS	9.92	(2.02)	10.00	(1.65)	0.874	
Muscle strength (kg)						
righ	45.21	(11.62)	45.65	(12.83)	0.617	
left	41.78	(11.40)	41.30	(10.82)	0.626	
30s-CST, *n (%)*	16.17	(4.67)	20.33	(5.42)	0.019	0.79
Functional Capacity*, mean (SD)						
Time (min)	10.28	(2.81)	10.94	(3.78)	0.387	
VO_2_ (mL/kg/min)						
in VT1	13.17	(8.32)	14.60	(10.83)	0.121	
in VT2	19.08	(8.79)	32.11	(34.89)	0.304	
maximum	22.66	(9.19)	23.67	(11.95)	0.395	
HR (bpm)						
in VT1	86.18	(17.81)	94.00	(18.99)	0.035	0.39
in VT2	112.45	(21.71)	113.56	(21.85)	0.076	
maximum	124.82	(20.89)	125.67	(18.84)	0.274	
Power (W)						
in VT1	65.73	(55.64)	87.89	(68.99)	0.012	0.35
in VT2	121.91	(80.71)	137.00	(84.72)	0.814	
maximum	143.91	(82.38)	159.89	(80.11)	0.310	
*Physical activity level (IPAQ), n (%)*						
Low	0	(0.00)	0	(0.00)	0.375	
Moderate	8	(66.70)	5	(41.70)		
Vigorous	4	(33.30)	7	(58.30)		

a, Chi-square test; *: ergospirometry; SD: standard deviation, Cohen’s d: moderate effect size; HR: heart rate; HADS: Hospital Anxiety and Depression Scale; HDL: high-density lipoprotein; IPAQ: International Physical Activity Questionnaire; lpm: beats per minute; LDL: low-density lipoprotein; MEDAS: questionnaire on adherence to the Mediterranean diet; CRP: cardiac rehabilitation program; 30s-CST: 30 second chair stand test; VO_2_, oxygen consumption; VT1, aerobic threshold; VT2: anaerobic threshold.

Neither group showed significant changes in heart rate, blood pressure, weight, abdominal circumference, anxiety, depression, smoking, and adherence to the medical diet.

If we compare the significant changes experienced by both groups at the end of the CRP with respect to their baseline situation, the out-of-hospital CRP group increased the mean HDL cholesterol (11.0 vs 0.63 mg/dL) more, while the heart rate in VT2 increased more in patients with in-hospital CRP (11.17 vs 2.88 bpm). No differences were found between groups in the variables of oxygen consumption (Table 4).

**Table 4:** Comparison of the differences observed in both groups

	Cardiac Rehabilitation Program		p
	Hospital	Out of hospital	
	n = 10	n = 12	(t-test)
*Clinical and anthropometric variables, mean (SD)*			
Heart rate (lpm),	-6.25	-3.17	0.689
Systolic blood pressure (mm Hg)	8.50	6.16	0.760
Weight (kg)	-0.73	0.11	0.490
Abdominal circumference (cm)	-1.00	-0.40	0.822
*Biochemical parameters, mean (SD)*			
Total cholesterol (mg/dL)	-23.67	-5.80	0.264
LDL (mg/dL)	-23.50	-12.20	0.449
HDL (mg/dL)	0.63	11.00	0.019
Triglycerides (mg/dL)	8.89	-24.60	0.072
Glucose (mg/dL)	-6.44	-0.40	0.517
% Glycated hemoglobin	-0.70	0.15	0.065
*Psychological variables (HADS), n (%)*			
Anxiety (*end*)	1 (14.3)	3 (14.3)	0.594
Depression (*end*)	0 (0.0)	0 (0.0)	1
*Questionnaires (score), mean (SD)*			
Fagerström	0.00	-0.08	0.461
Richmond	1.40	-0.42	0.143
MEDAS	0.85	0.08	0.433
Muscle strength (kg)			
right	-2.07	0.44	0.158
left	-1.16	-0.48	0.715
30s-CST, *n (%)*	6.86	4.17	0.292
Functional capacity*, mean (SD)			
Time (min)	0.57	0.80	0.854
VO_2_ (mL/kg/min)			
in VT1	0.58	1.93	0.349
in VT2	0.13	-14.43	0.357
maximum	-1.83	1.45	0.170
HR (bpm)			
in VT1	10.83	9.75	0.822
in VT2	11.17	2.88	0.039
maximum	3.67	6.25	0.708
Watts (W)			
in VT1	19.5	18.63	0.924
in VT2	19.33	1.50	0.099
maximum	8.33	10.00	0.886
*Physical activity level (IPAQ), n (%)*			
Low (*final*)	0 (0.0)	0 (0.0)	1
Moderate (*final*)	5 (71.4)	5 (41.7)	1
Vigorous (*final*)	2 (28.6)	7 (58.3)	0.099

*: ergospirometry; SD: standard deviation; Cohen’s d: moderate effect size; HR: heart rate; HADS: Hospital Anxiety and Depression Scale; HDL: high-density lipoprotein; IPAQ: International Physical Activity Questionnaire; lpm: beats per minute; LDL: low-density lipoprotein; MEDAS: questionnaire on adherence to the Mediterranean diet; CRP: cardiac rehabilitation program; 30S-CST: 30 second chair stand test; VO2, oxygen consumption; VT1, aerobic threshold; VT2: anaerobic threshold.

## DISCUSSION

This study compares the effects of a CRP performed in a municipal sports center with respect to a hospital CRP on compliance, functional capacity, and different variables (clinical, anthropometric, biochemical, psychological, physical, and habits). Although it yields interesting results, these are biased by a lack of statistical power due to the small sample size derived from the state of emergency due to COVID-19 and, therefore, does not allow us to demonstrate the real impact of an out-of-hospital CRP in a municipal sports center.

No examples of out-of-hospital CRPs performed in sports centers were found in the literature. Most studies analyze home CRPs and describe improvements in oxygen consumption, perceived social support, quality of physical life^28^^,^^29^, functional capacity^30^ and levels of anxiety and depression^31^, unlike our study where we found no improvements in these variables.

Although we have not found statistically significant differences in levels of physical activity within and between groups, recent studies in patients with coronary heart disease have demonstrated the efficacy of cardiac rehabilitation interventions in improving physical activity, physical fitness, functional capacity and reducing sedentary lifestyles^32^^,^^33^.

The changes observed in the lipid profile of the patients with respect to the improvement of HDL levels in the out-of-hospital group are consistent with the results obtained in hybrid models of rehabilitation in patients with coronary disease^33^^,^^34^ or other pathologies, such as heart failure, where the CRPs are based on patient education in the control of cardiovascular risk factors and in the development of a lifestyle have demonstrated broad beneficial effects on health status and quality of life^3^^,^^5^^,^^35^. However, this effect should be taken with caution since HDL levels are significantly lower at baseline in the out-of-hospital group, which may bias the results of significant improvement.

This study has tried to respond to a real problem in our environment: the contrast between the high demand for hospital CRP places and the high demand for hospital CRP places in the hospital and the high demand for hospital places in the hospital.

The limited number of places available in hospitals. Alternatives to conventional cardiac rehabilitation in hospitals, by providing safe spaces in municipal sports centers, could help to reduce this limitation, allowing access to all patients and reserving hospital places for patients at higher risk. In addition, the opening hours of the municipal sports center in this study (morning and afternoon) were greater than those available in the cardiac rehabilitation unit of the hospital (morning only), so the implementation of this out-of-hospital CRP would make it easier to adapt to the social, family and work life of the patients. However, the effectiveness of this type of CRP could not be proven with the data from this study.

The out-of-hospital CRP was carried out with the participation of a physiotherapist belonging to the research team. However, there are municipal sports centers that have sports medical centers, sports doctors, nurses, and physiotherapists, to whom patients could be referred from hospital cardiac rehabilitation units to perform PEPs and thus facilitate access to cardiac rehabilitation.

The health education program is carried out in the hospital’s main classrooms and is open to any patient with cardiovascular disease and their families, which allows patients referred from primary care and those participating in out-of-hospital rehabilitation programs to attend. Access to physical activity monitoring devices and mobile applications, which are part of the remote intervention, can complement the physical exercise program and indications administered in rehabilitation, and their use has been shown to reduce the risk of mortality and rehospitalization in the context of rehabilitation^36^. Mobile applications can complement or cover the educational aspects addressed in both hospital and out-of-hospital CRPs, facilitating the implementation of out-of-hospital CRPs in a safe manner in the primary care environment, under the supervision of the primary doctor and their coordination with municipal sports centers. Given that the economic impact of this model of CRP is unknown, it would be interesting to carry out studies in our environment that would analyze it.

Due to the social situation resulting from the COVID-19 pandemic, this study had to be interrupted early and some patients could not be followed up and excluded from the analysis. In addition, the interruption of the inpatient cardiac rehabilitation program and the closure of municipal sports centers prevented the completion of the full recruitment of patients, which is the main limitation of this study. This situation has resulted in a loss of statistical power due to the small sample size. In addition, this is a single-center study, and the data may not be generalizable to other populations or areas of health care. As it was a non-blind design, the participants were aware of the group they belonged to, so this effect may have interfered with the results obtained. On the other hand, no post-completion follow-up of CRPs was performed to see long-term effects. Due to the waiting list at our center, participants started CRPs six months after hospital discharge, a situation that may not be generalizable to other centers with different waiting lists. For this reason, it would be advisable to carry out multicenter studies, with larger sample sizes and long-term follow-ups, in order to contrast the results of this study and expand knowledge about the benefits of telerehabilitation in municipal sports spaces.

By way of conclusions, this study has not been able to determine the efficacy of out-of--hospital CRPs due to lack of potency (abundant absences due to COVID-19 confinement). Regarding the differences analyzed between groups, significant increases in HDL cholesterol levels were only observed in patients who underwent out-of-hospital CRP, but these results may be biased since the groups did not have the same baseline levels, while patients who underwent in-hospital CRP significantly increased heart rate in VT2. Studies should be carried out to study the feasibility of out-of-hospital non-home cardiac rehabilitation services to care for these patients with mild or moderate coronary involvement.
